# How generative AI shapes designers’ cultural heritage revitalization intention: process and content cues in AI-assisted cultural heritage design

**DOI:** 10.3389/fpsyg.2026.1904639

**Published:** 2026-07-16

**Authors:** Chang Liu, Ping Cang

**Affiliations:** 1 College of Fashion and Design, Donghua University, Shanghai, China; 2 Shanghai Urban Construction Vocational College, Shanghai, China

**Keywords:** cultural heritage revitalization, cultural sustainability, designer experience, fsQCA, generative artificial intelligence, human—AI collaboration, perceived output quality, psychological ownership

## Abstract

As generative AI becomes increasingly involved in cultural heritage design, designers’ evaluations of AI collaboration are shaping cultural revitalization practices. Yet existing research offers limited explanation of how AI characteristics affect designers’ willingness to engage in cultural heritage revitalization or how professional experience conditions this process. Based on the stimulus-organism-response (S-O-R) framework, this study develops a dual-path moderated mediation model. It examines how AI process cues and AI-generated content cues influence revitalization intention through psychological ownership and perceived output quality. Using data from 312 Chinese designers with experience in generative AI-assisted cultural heritage design, the study combines PLS-SEM, multi-group analysis, fsQCA, and LDA text analysis. The results show that algorithmic autonomy reduces psychological ownership, whereas interaction transparency and interaction depth enhance it. Cultural authenticity improves perceived output quality, while the effect of visual complexity varies by experience level. Experienced designers rely more on psychological ownership, whereas early-career designers rely more on perceived output quality. fsQCA and LDA further reveal multiple configurations of high revitalization intention and two evaluative orientations in design discourse: ownership-oriented and quality-oriented. This study clarifies the dual-evaluation mechanism of AI-assisted cultural heritage design and offers behavioral evidence for generative AI’s role in cultural translation and sustainable dissemination.

## 1 Introduction

Cultural heritage is a key cultural resource formed through human societies’ long-term adaptation to natural environments, social orders, and collective life (Hu et al., 2024). In the context of sustainable development, artificial intelligence (AI) technologies are increasingly used in cultural heritage protection, reconstruction, restoration, and dissemination. These applications support sustainable preservation and intergenerational transmission (Laohaviraphap and Waroonkun, 2024; Min, 2025; Mishra and Lourenço, 2024). The role of new technologies in the contemporary expression of cultural heritage has therefore become a shared concern in cultural studies, design research, and sustainability studies.

Generative AI tools such as Midjourney, DALL-E, and Stable Diffusion differ from traditional computer-aided design tools. They improve visual generation efficiency and participate in the identification, recombination, and re-expression of cultural symbols (Zhang et al., 2023; Zhou and Lee, 2024). In AI-assisted cultural heritage design, algorithmic systems are shifting from technical tools to intelligent media involved in cultural expression (Atkinson and Barker, 2023; McGuire et al., 2024). This shift may further influence designers’ judgments of cultural authenticity and value (Bui et al., 2024; Messer, 2024). Yet AI involvement does not necessarily lead to effective cultural heritage revitalization. When generated solutions merely assemble cultural elements and still gain market or platform recognition, designers may question their cultural authenticity and professional value. This may weaken their willingness to continue engaging in AI-assisted cultural revitalization (Zhang et al., 2024). It is therefore necessary to examine how AI characteristics affect designers’ evaluations of the collaborative process and generated outcomes, and how these evaluations shape their revitalization intention. This issue concerns the practical effectiveness of AI-assisted design and the continued production and circulation of cultural meaning in digital environments.

Existing studies have examined AI and cultural design from several perspectives. Early research treated AI mainly as a technical means for digital cultural heritage preservation. It focused on recognition, classification, restoration, and recommendation systems, and emphasized AI’s efficiency in organizing, protecting, and presenting cultural resources (Gîrbacia, 2024; Lai et al., 2025). Recent studies have turned to cultural experiences and application scenarios, including museum scene design, cultural and creative product generation, and users’ purchase intentions (Dongjiao et al., 2025; Gurel, 2026; Shi et al., 2025). Research on generative AI design has also examined tool interaction, human-AI collaboration, and design performance (Bankins et al., 2024; Hou et al., 2025). These studies provide an important basis for understanding the application value of AI. However, from the perspective of cultural sustainability, AI-assisted cultural heritage design should not be understood only as a matter of efficiency improvement or process optimization. Whether cultural heritage can be continuously revitalized in digital environments also depends on how designers understand AI’s mode of participation, how they evaluate the cultural value of generated content, and whether they are willing to continue engaging in cultural revitalization practices.

Recent research shows that AI collaboration experiences may affect designers’ psychological states, and that design experience may change this mechanism (Gong and Chen, 2026; Rafner et al., 2025). Nevertheless, these discussions still focus mainly on creativity, control allocation, and design performance in general design contexts (Huang et al., 2026). In cultural heritage design, the key issue extends beyond whether AI can improve efficiency to whether designers recognize its cultural and professional value. If designers do not accept AI’s mode of participation or generated outcomes, AI is unlikely to support sustained cultural revitalization. Based on this reasoning, this study examines how different AI characteristics influence designers’ evaluations of the collaborative process and generated outcomes, and how these evaluations affect their willingness to engage in cultural heritage revitalization. In this study, “cultural heritage” refers to cultural resources characterized by historical continuity, collective identity, and symbolic significance, and incorporated into generative AI-assisted design processes. These resources include both tangible forms and intangible cultural content. Although differences in heritage type and modes of preservation are acknowledged, the analysis focuses on how these resources contribute to cultural symbol generation, design alternative selection, and authenticity assessment within AI-assisted design. Accordingly, heritage typology is not treated as the primary analytical dimension.

Overall, three research gaps remain. First, AI process characteristics and AI-generated content characteristics have not been systematically integrated into a single analytical framework. When designers use AI for cultural heritage design, they evaluate how algorithms participate in creation and whether the generated content has cultural and professional value. Second, although AI is increasingly used for cultural heritage revitalization, empirical explanations remain limited regarding how designers with different levels of experience respond to AI involvement. Third, existing studies often rely on a single method, which limits their ability to reveal average effects, group differences, and complex configurations of conditions at the same time. To address these gaps, this study proposes the following research questions: (1) How do AI process characteristics, including algorithmic autonomy, interaction transparency, and interaction depth, affect designers’ psychological ownership? (2) How do AI-generated content characteristics, including cultural authenticity and visual complexity, affect designers’ perceived output quality? (3) How do psychological ownership and perceived output quality influence willingness to engage in cultural heritage revitalization, and does this mechanism vary according to designers’ professional experience?

This study adopts a stimulus-organism-response (S-O-R) framework from the perspective of AI characteristics. AI process characteristics and AI-generated content characteristics are treated as external stimuli. Psychological ownership and perceived output quality are treated as designers’ internal psychological and evaluative states. Willingness to engage in cultural heritage revitalization is treated as the behavioral response. Professional experience is introduced as a moderating boundary condition to examine differences between senior designers and early career designers. Methodologically, this study combines partial least squares structural equation modeling (PLS-SEM), multi-group analysis, fuzzy-set qualitative comparative analysis (fsQCA), and latent Dirichlet allocation (LDA) topic modeling. PLS-SEM tests the structural paths. Multi-group analysis compares differences between experience groups. fsQCA identifies configurations associated with high willingness to engage in cultural heritage revitalization. LDA provides supplementary evidence from professional and public design contexts.

The research makes three contributions. First, it integrates AI process characteristics and AI-generated content characteristics into a single analytical framework, showing that AI-assisted cultural heritage revitalization is jointly shaped by the collaborative process and generated outcomes. Second, it extends the outcome variable from general design creativity or technology use intention to willingness to engage in cultural heritage revitalization, thereby highlighting the importance of cultural authenticity, output quality, and designers’ subjective judgment. Third, it reveals the boundary role of professional experience in AI-assisted cultural heritage design, providing empirical evidence for cultural institutions, design teams, and AI platforms to develop differentiated collaboration strategies.

## 2 Literature review and hypotheses

### 2.1 AI-assisted cultural heritage design as a dual-evaluation process

The difficulty of cultural heritage design lies not in the use of traditional symbols *per se*, but in whether these symbols can retain identifiable cultural origins, meanings, and contemporary value when placed in new design contexts. Designers must extract meaningful forms and cultural values from cultural carriers and translate them into products or services that contemporary users can understand, use, and disseminate (Xiang et al., 2025; Zhu and Rahman, 2025). Thus, cultural heritage design should be evaluated in terms of novelty or visual appeal as well as by whether it conveys cultural meaning accurately and fits new contexts of use (Yin et al., 2026).

Generative AI changes this translation process. Unlike traditional CAD tools, which mainly support drawing, modeling, and visual refinement, generative AI can assist in concept generation, image production, solution exploration, and iterative modification (Saadi et al., 2024; Verganti et al., 2020). Designers therefore increasingly collaborate with AI through prompting, feedback, selection, and adjustment, rather than producing visual solutions entirely by themselves (Rezwana and Maher, 2023). In human-AI co-creation, the final output is not the only object of evaluation; interaction patterns, feedback structures, and collaborative iterations also shape the allocation of creative responsibility (Rafner et al., 2025). This is especially relevant to cultural heritage design, where designers act as tool users, interpreters of cultural meaning, and gatekeepers of symbolic appropriateness.

AI-assisted cultural heritage design can therefore be understood as a dual-evaluation process. Designers first evaluate how AI participates in creation, including whether the algorithm is overly autonomous, whether its generation logic is understandable, and whether interaction allows continuous revision and redirection. Such process evaluation affects whether designers feel they can still influence the final outcome. Designers also evaluate what AI generates, including whether the output preserves cultural authenticity and whether it has visual completeness, professional usability, and value for further development. This content evaluation differs from aesthetic preference alone: an image may be visually refined yet still involve cultural collage, symbolic misinterpretation, or contextual loss (Farrelly et al., 2019; Paolanti et al., 2026).

This study uses the S-O-R framework to explain the dual evaluative process through which designers respond to generative AI in cultural heritage design. Research based on the S-O-R framework suggests that external stimuli influence behavioral responses through individuals’ internal psychological and evaluative states (Mehrabian and Russell, 1974; Nian et al., 2025). Recent work on generative AI further shows that this framework can explain how perceived AI-related cues shape internal states and behavioral intention in AI adoption contexts (Dalvi-Esfahani et al., 2025). Generative AI differs from conventional design tools because it actively generates, revises, and expands design possibilities, thereby reshaping the distribution of agency between humans and AI in creative work (Krakowski, 2025; Wessel et al., 2025). In text-to-image visual design, prompting, generation, feedback, and iteration expose designers to two interrelated sources of evaluation: the way AI participates in the collaborative process and the qualities shown in AI-generated outputs (Herath et al., 2026). The first concerns creative decision authority, the intelligibility of generative logic, and opportunities for continued revision. These aspects affect designers’ sense of control, personal investment, and authorship, and are therefore linked to psychological ownership. The second concerns the cultural value, formal completeness, and professional usability of generated outputs. These aspects shape designers’ judgments of output quality and its potential for further development. Together, psychological ownership and perceived output quality influence designers’ willingness to continue using AI for cultural translation, design development, and cultural heritage revitalization.

### 2.2 AI process characteristics

#### 2.2.1 Algorithmic autonomy

Algorithmic autonomy refers to the extent to which AI can make complex creative decisions with limited human intervention (Fan and Liu, 2022). Research on generative AI and ownership perception suggests that algorithmic replacement of decision authority can weaken creators’ sense of authorship and creative identity (Hermann, 2025; Lee et al., 2026). When creative authority is transferred to the algorithm, designers may perceive weaker influence over the final output, which can erode their sense of agency (Chen and He, 2026; Elias et al., 2025). Therefore, this study argues that when algorithms show a high level of independent decision-making capacity, designers’ psychological ownership will be inhibited. The following hypothesis is proposed:

*H1:* AI algorithmic autonomy negatively affects designers’ psychological ownership.

#### 2.2.2 Interaction transparency

Interaction transparency refers to the explainability of an algorithm’s generative logic (Shin, 2021). In human–AI collaboration, transparent interaction mechanisms make algorithmic decision paths more understandable and allow designers to monitor and adjust the generation process (Mueller et al., 2019; Rajabi and Etminani, 2024). Prior studies show that explainable AI can strengthen user trust and improve the perceived quality of explanations, thereby restoring a sense of control that is central to psychological ownership (Kim et al., 2024; Xu and Wang, 2024). This study argues that interaction transparency helps designers maintain perceived influence over AI-assisted outputs. When designers can understand and adjust the generation process, they are more likely to perceive the output as shaped by their own judgment. Since perceived control is central to psychological ownership, interaction transparency is expected to strengthen designers’ psychological ownership. Accordingly, the following hypothesis is proposed:

*H2:* Interaction transparency positively affects designers’ psychological ownership.

#### 2.2.3 Interaction depth

Interaction depth refers to the frequency of prompt refinement and design iteration between designers and AI systems before a final output is produced (Park and Choo, 2025). It reflects the degree of cognitive investment in the collaborative process (Oppenlaender et al., 2025). Psychological ownership theory suggests that individuals develop a stronger sense of ownership when they invest more time and creative energy in a target (Blaurock et al., 2025; Jiang et al., 2025). Repeated interaction allows designers to continuously embed their intentions into the output, thereby strengthening their psychological attachment to it. Accordingly, the following hypothesis is proposed:

*H3:* Interaction depth positively affects designers’ psychological ownership.

### 2.3 AI-generated content characteristics

AI-generated content characteristics refer to the output cues that designers perceive in generated results (Zhou et al., 2024). In cultural heritage design, such outputs are design outcomes produced through the generative AI translation of cultural resources. Their evaluation therefore involves both cultural meaning and visual form. Prior design research suggests that when cultural resources enter design practice, cultural knowledge, artifact features, and symbolic meanings must be translated into operable design language (Dong et al., 2023; Leong and Clark, 2003). Accordingly, cultural authenticity concerns the cultural validity of generated content, including cultural origin, contextual relations, semantic coherence, and symbolic appropriateness. Visual complexity concerns its formal developability, including visual detail, formal richness, and completeness (Van Geert and Wagemans, 2020). The former addresses whether the output is culturally grounded; the latter addresses whether it is formally ready for further design development.

#### 2.3.1 Cultural authenticity

Cultural authenticity refers to the extent to which AI-generated content maintains the cultural meanings, origins, and symbolic appropriateness of heritage resources (Paolanti et al., 2026). In cultural heritage preservation and design translation, authenticity is widely treated as a central criterion for evaluating design outputs (Farrelly et al., 2019; McIntosh and Prentice, 1999). Authentic outputs can reduce concerns about cultural misrepresentation and strengthen designers’ recognition of a work’s professional value. Therefore, this study treats cultural authenticity as a key antecedent of perceived output quality (Gangadharbatla, 2022; Jacobs et al., 2025). Thus, the following hypothesis is proposed:

*H4:* The cultural authenticity of generated content positively affects designers’ perceived output quality.

#### 2.3.2 Visual complexity

Visual complexity refers to the formal refinement, visual detail, and compositional richness of generated images (Wals et al., 2026). Prior research suggests that generative technologies are effective in producing detailed images and complex visual symbols (Brachmann and Redies, 2017; Zhang et al., 2021). Visual richness can produce immediate aesthetic pleasure and raise designers’ evaluations of the artistic quality of digital works (Guo et al., 2018). In cultural heritage redesign, visual complexity functions as a cue of formal completeness and design developability (Chen S. et al., 2025; Min et al., 2025). It helps designers judge whether a generated output has sufficient visual organization, detail density, and structural richness to support subsequent product, communication, or experience design (Chen J. et al., 2025). This study therefore expects visual complexity to improve perceived output quality. The following hypothesis is proposed:

*H5:* The visual complexity of generated content positively affects designers’ perceived output quality.

### 2.4 Internal states and revitalization intention

Psychological ownership refers to a state in which individuals perceive a target as an extension of the self. It is formed through perceived control, self-investment, and intimate knowledge of the target (Pierce et al., 2003). In AI collaboration, this sense of ownership can shape how individuals evaluate AI-generated outputs (Yang et al., 2025; Zhang and Hu, 2024). When designers perceive greater interaction transparency and deeper iteration, collaboration can restore a sense of control and create cognitive self-investment. This may reduce the alienation caused by algorithmic automation (Shin, 2021). As designers begin to view an algorithmic output as their own creative work, they may develop stronger professional responsibility and protection motivation, which in turn supports their intention to engage in cultural heritage revitalization (Kim et al., 2025; Mukhtar et al., 2026). Psychological ownership is therefore treated as an internal mechanism linking AI process characteristics to revitalization intention.

*H6:* Psychological ownership mediates the relationship between AI process characteristics, namely algorithmic autonomy, interaction transparency, and interaction depth, and cultural heritage revitalization intention.

Perceived quality refers to an evaluator’s subjective judgment of an object’s overall excellence and suitability based on personal experience, needs, and context (Zeithaml, 1988). When direct assessment is difficult, evaluators often rely on observable cues to infer quality (Olson and Jacoby, 1972). In AI-assisted cultural heritage design, cultural authenticity and visual complexity can serve as two key content cues for perceived output quality. Cultural authenticity helps designers assess whether the generated output preserves cultural context and symbolic meaning. Visual complexity helps them assess whether the output shows visual refinement and design completeness (Farrelly et al., 2019; Wals et al., 2026). Cultural authenticity may strengthen designers’ recognition of the cultural value and professional appropriateness of the output (Kreuzbauer and Keller, 2017). Visual complexity provides a basis for judging image completeness and potential for further development (Baek et al., 2023). When designers perceive AI-generated outputs as high in quality, they are more likely to regard them as design resources for cultural translation, communication design, and product development (Fu and Razmjooy, 2025; Liang, 2024). Perceived output quality can therefore function as an evaluative mechanism linking AI-generated content characteristics to willingness to engage in cultural heritage revitalization.

*H7:* Perceived output quality mediates the relationship between AI-generated content characteristics, namely cultural authenticity and visual complexity, and cultural heritage revitalization intention.

### 2.5 The role of professional experience

Professional experience shapes how designers process design information and judge design value. Cognitive psychology research shows that experience level affects knowledge representation, attention allocation, and information integration. Designers with different levels of experience may therefore attend to different cues when evaluating the same complex object (Chen et al., 2022; Tan, 2021). In the design field, experienced designers often rely on more stable knowledge schemas and evaluative criteria to organize design solutions. Early career designers are more likely to judge solution value through external feedback and visible outcomes (Ozkan and Dogan, 2013). In AI-assisted cultural heritage design, these experience-based differences affect the relative importance of two internal states. For experienced designers, psychological ownership better reflects their recognition of the collaborative process, creative influence, and authority in cultural judgment (Gong and Chen, 2026; Pierce et al., 2001). For early career designers, perceived output quality is more directly linked to whether AI provides usable, complete, and developable design outcomes (Ahmed et al., 2003; Li, 2025). Professional experience therefore moderates the effects of psychological ownership and perceived output quality on willingness to engage in cultural heritage revitalization. Based on this reasoning, the following hypotheses are proposed:

*H8:* Designers’ professional experience moderates the effects of psychological ownership and perceived output quality on revitalization intention.

*H8a:* The effect of psychological ownership on revitalization intention is stronger among experienced designers than among early career designers.

*H8b:* The effect of perceived output quality on revitalization intention is stronger among early-career designers than among experienced designers.

**FIGURE 1:**
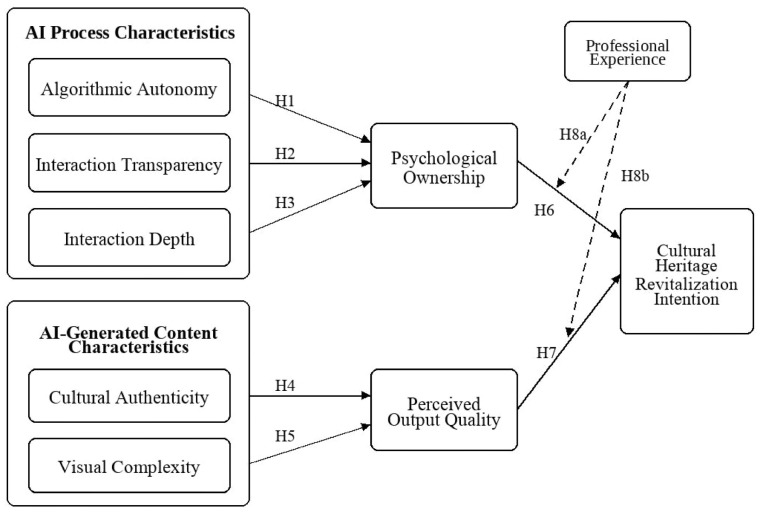
Research model.

Figure 1 presents the theoretical framework of this study.

## 3 Materials and methods

### 3.1 Measurement items

All constructs were measured using established scales adapted from prior validated studies. Algorithmic autonomy was adapted from Parasuraman et al. (2000) work on automation and Shin’s measure of AI autonomy (Shin, 2021). Interaction transparency was adapted from Shin (2021) and Kizilcec (2016) scales of algorithmic transparency. Interaction depth was adapted from O’Brien et al. (2018) User Engagement Scale (UES), with a focus on cognitive immersion and iteration frequency in collaboration. For AI-generated content characteristics, cultural authenticity was adapted from Kolar and Zabkar’s cultural heritage authenticity scale (Kolar and Zabkar, 2010), and visual complexity was adapted from Pieters et al. (2010). Psychological ownership was adapted from Pierce et al. (2001) and Avey et al. (2009). Perceived output quality was adapted from Zeithaml (1988) and Dodds et al. (1991). Cultural heritage revitalization intention was developed with reference to Ajzen’s theory of planned behavior (Ajzen, 1991), with wording adjusted to the context of cultural heritage protection and communication.

All items were measured on a seven-point Likert scale, ranging from strongly disagree (1) to strongly agree (7). Before the formal survey, the questionnaire was reviewed by three experts in human–computer interaction and two senior designers. A pilot test was then conducted with 50 distributed questionnaires, of which 46 were valid, giving a valid response rate of 92%. Based on the pilot results, items with ambiguous wording or unclear logic were revised. The final questionnaire is reported in Table 1, including the measurement items, variable definitions, and original scale sources.

**TABLE 1:** Variables, measurement items, and references.

Variable	Measurement items	References
Algorithmic autonomy	AA1. The AI system can make key creative decisions without my detailed guidance. AA2. The AI system can independently determine the composition and color scheme of the design. AA3. The AI system can largely determine the final direction of the design.	(Parasuraman et al., 2000; Shin, 2021)
Interaction transparency	IT1. I understand how the AI system generates designs based on my prompts. IT2. The system provides understandable logic for its generated outputs. IT3. The interaction process makes the AI system’s decision-making process visible to me.	(Kizilcec, 2016; Shin, 2021)
Interaction depth	ID1. I invested substantial cognitive effort in iterating the design with the AI system. ID2. I spent considerable time refining prompts to improve the design outcome. ID3. The collaboration involved multiple rounds of prompt adjustment and output revision.	(O’Brien et al., 2018)
Cultural authenticity	CA1. The AI-generated content reflects the core meanings of cultural heritage. CA2. The generated content is consistent with the logic and symbolic system of traditional culture. CA3. The design output preserves the cultural characteristics of the heritage source.	(Kolar and Zabkar, 2010)
Visual complexity	VC1. The generated patterns contain rich visual details. VC2. The design output shows a high level of visual refinement. VC3. The design output presents layered visual information.	(Pieters et al., 2010)
Psychological ownership	PO1. I feel that the AI-generated design work is mine. PO2. I feel a sense of ownership over the final collaborative design. PO3. I feel psychologically connected to the work created with the AI system.	(Avey et al., 2009; Pierce et al., 2001)
Perceived output quality	OQ1. The design outputs generated with the AI system are of high quality. OQ2. The AI-generated outputs meet professional design standards. OQ3. The final design output is suitable for professional design practice.	(Dodds et al., 1991; Zeithaml, 1988)
Cultural heritage revitalization intention	RI1. I am willing to use AI tools to transform traditional cultural heritage elements into design outputs suited to contemporary contexts. RI2. I intend to use AI-assisted tools to support the contemporary communication of cultural heritage. RI3. I will invest more effort in exploring new possibilities for cultural heritage revitalization through AI.	(Ajzen, 1991)

### 3.2 Data collection

Participants were recruited through design communities and design groups at universities. The study used purposive and snowball sampling to reach designers with experience using generative AI. The first page of the questionnaire presented an informed consent statement, which described the research purpose, data use, anonymity protections, and participants’ right to withdraw at any time. Only participants who selected the consent option were directed to the formal survey; those who declined were exited from the questionnaire. Eligibility was defined by three criteria: being at least 18 years old, having a design-related educational or professional background, and having recently used a generative AI tool in a cultural heritage-related design task. A screening item confirmed eligibility before entry into the formal questionnaire. Before completing the focal scales, eligible respondents were asked to recall a representative AI-assisted cultural heritage design task. This retrospective prompt provided a concrete reference for evaluating AI process characteristics and AI-generated content characteristics.

To examine the moderating role of professional experience, respondents were classified into early career and experienced designer groups according to years of design-related experience and professional background. This operational distinction follows prior design expertise research, which differentiates novice and experienced designers based on accumulated design experience and differences in design-task approaches (Ahmed et al., 2003; Kalali Sani et al., 2022). After removing invalid responses with very short completion times or patterned answers, 312 valid responses were retained from 420 collected responses. Senior designers accounted for 40.7% of the sample, and junior designers and students in related fields accounted for 59.3%. Table 2 reports the demographic and professional profiles of the respondents.

**TABLE 2:** Descriptive statistics of the valid sample.

Sample characteristic	Category	Frequency	Percentage (%)
Gender	Female	165	52.9
	Male	147	47.1
Age	18–24 years	150	48.1
	25–34 years	110	35.3
	35–45 years	32	10.3
	46 years or above	20	6.4
Design experience group	Early career group: 5 years or less	185	59.3
	Experienced group: 6 years or more	127	40.7
Frequently used AI tool	Midjourney	115	36.9
	Nano banana	92	29.5
	Adobe firefly	55	17.6
	Other tools	50	16.0
Weekly AI collaboration time	< 5 h	85	27.2
	5–15 h	155	49.7
	More than 15 h	72	23.1

## 4 Results

### 4.1 Measurement model assessment

Data were analyzed using partial least squares structural equation modeling (PLS-SEM) (Hair et al., 2019). Before testing the structural paths, the measurement model was assessed for reliability, convergent validity, and discriminant validity. Convergent validity was examined using composite reliability (CR) and average variance extracted (AVE). As reported in Table 3, all item loadings were above 0.70. The AVE values exceeded 0.60, and the CR values exceeded 0.80, indicating adequate convergent validity (Bagozzi and Yi, 1988). Cronbach’s α values were also above 0.70 for all constructs, supporting internal consistency reliability.

**TABLE 3:** Reliability and convergent validity of the measurement scales.

Construct	Cronbach’s α	Composite reliability (CR)	Average variance extracted (AVE)
Algorithmic autonomy	0.877	0.924	0.802
Interaction transparency	0.870	0.920	0.794
Interaction depth	0.859	0.914	0.780
Cultural authenticity	0.843	0.905	0.761
Visual complexity	0.874	0.922	0.798
Psychological ownership	0.861	0.915	0.783
Perceived output quality	0.864	0.917	0.786
Cultural heritage revitalization intention	0.872	0.922	0.797

Discriminant validity was assessed using the Fornell–Larcker criterion (Fornell and Larcker, 1981). As shown in Table 4, the square root of AVE for each construct was higher than its correlations with the other constructs, supporting discriminant validity.

**TABLE 4:** Discriminant validity based on the Fornell–Larcker criterion.

Construct	X1	X2	X3	X4	X5	X6	X7	Y
Algorithmic autonomy (X1)	**0.896**	**0.891**	**0.883**	**0.873**	**0.894**	**0.885**	**0.887**	**0.893**
Interaction transparency (X2)	0.244							
Interaction depth (X3)	0.264	0.334						
Cultural authenticity (X4)	0.177	0.210	0.280					
Visual complexity (X5)	0.217	0.304	0.274	0.173				
Psychological ownership (X6)	-0.329	0.296	0.288	0.262	0.233			
Perceived output quality (X7)	0.246	0.255	0.372	0.297	0.285	0.256		
Cultural heritage revitalization intention (Y)	0.235	0.248	0.355	0.193	0.250	0.277	0.346	

Bold diagonal values are the square roots of AVE.

Discriminant validity was further assessed using the heterotrait–monotrait ratio (HTMT), which provides a more stringent assessment of construct distinctiveness than traditional criteria *(Henseler et al., 2015; Voorhees et al., 2016). As reported in Table 5, all HTMT values met the recommended criterion, providing additional support for the discriminant validity of the measurement model.*

**TABLE 5:** Heterotrait-monotrait ratio (HTMT) matrix.

Construct	AA	IT	ID	CA	VC	PO	OQ	RI
AA	0.280	0.386	0.330	0.201	0.268	0.297	0.399	
IT								
ID	0.304							
CA	0.206	0.245						
VC	0.248	0.348	0.316					
PO	0.379	0.342	0.335	0.307				
OQ	0.283	0.294	0.431	0.348	0.328			
RI	0.268	0.285	0.411	0.224	0.286	0.319		

### 4.2 Common method bias

To assess multicollinearity and common method bias (CMB), variance inflation factors (VIFs) were first examined. The VIF values ranged from 1.450 to 2.102, below the recommended threshold of 3, suggesting no serious multicollinearity problem (Hair et al., 2019). Harman’s single-factor test was then conducted. The first unrotated factor explained 27.365% of the total variance, below the 40% threshold, indicating that CMB was unlikely to be a major concern (Podsakoff et al., 2003). Because Harman’s test has known limitations, a marker-variable test was also performed. Age and gender were used as marker variables because they had limited theoretical relevance to the focal constructs. After including these marker variables, the significance of the main path coefficients remained substantively unchanged, further suggesting that CMB did not seriously bias the results.

### 4.3 Structural model assessment

The hypotheses were tested using PLS-SEM in SmartPLS, with separate analyses conducted for each group. Bootstrapping was performed with 95% confidence intervals. In the experienced designer group, AI process characteristics were significant predictors of psychological ownership. Algorithmic autonomy negatively affected psychological ownership, supporting H1 (β = –0.431, *t* = 5.611, *p* < 0.001). Interaction transparency (β = 0.315, *t* = 4.084, *p* < 0.001) and interaction depth (β = 0.312, *t* = 4.746, *p* < 0.001) both had positive effects on psychological ownership, supporting H2 and H3. For AI-generated content characteristics, cultural authenticity positively affected perceived output quality, supporting H4 (β = 0.254, *t* = 3.075, *p* = 0.002). Visual complexity, however, had no significant effect on perceived output quality in this group (β = 0.071, *t* = 0.783, *p* = 0.434), so H5 was not supported. Regarding revitalization intention, psychological ownership had a stronger positive effect (β = 0.366, *t* = 4.824, *p* < 0.001), whereas perceived output quality had a weaker but still significant effect (β = 0.161, *t* = 2.036, *p* = 0.042). Table 6 presents the path coefficients.

**TABLE 6:** Path coefficients for the experienced designer group.

Path	Coefficient	*T*-value	*P*-value	2.5% CI	97.5% CI
Algorithmic autonomy → psychological ownership	–0.431	5.611	0.000	–0.571	–0.281
Interaction transparency → psychological ownership	0.315	4.084	0.000	0.171	0.463
Interaction depth → psychological ownership	0.312	4.746	0.000	0.183	0.441
Cultural authenticity → perceived output quality	0.254	3.075	0.002	0.101	0.421
Visual complexity → perceived output quality	0.071	0.783	0.434	–0.147	0.253
Psychological ownership → revitalization intention	0.366	4.824	0.000	0.216	0.515
Perceived output quality → revitalization intention	0.161	2.036	0.042	0.008	0.314

In the early career designer group, the relationships between AI process characteristics and psychological ownership were consistent with those observed in the experienced designer group. Algorithmic autonomy had a significant negative effect on psychological ownership, supporting H1 (β = –0.535, *t* = 10.176, *p* < 0.001). Interaction transparency (β = 0.297, *t* = 4.759, *p* < 0.001) and interaction depth (β = 0.334, *t* = 4.807, *p* < 0.001) both had positive effects on psychological ownership, supporting H2 and H3. For AI-generated content characteristics, both cultural authenticity (β = 0.298, *t* = 4.259, *p* < 0.001) and visual complexity (β = 0.363, *t* = 5.369, *p* < 0.001) positively affected perceived output quality, supporting H4 and H5. For revitalization intention, perceived output quality had a significant positive effect, whereas psychological ownership was not significant. Perceived output quality had a positive effect on revitalization intention (β = 0.384, *t* = 6.700, *p* < 0.001), whereas psychological ownership had no significant effect (β = 0.098, *t* = 1.355, *p* = 0.175). Table 7 presents the path coefficients.

**TABLE 7:** Path coefficients for the early career designer group.

Path	Coefficient	*T*-value	*P*-value	2.5% CI	97.5% CI
Algorithmic autonomy → psychological ownership	–0.535	10.176	0.000	–0.637	–0.433
Interaction transparency → psychological ownership	0.297	4.759	0.000	0.170	0.416
Interaction depth → psychological ownership	0.334	4.807	0.000	0.200	0.475
Cultural authenticity → perceived output quality	0.298	4.259	0.000	0.163	0.437
Visual complexity → perceived output quality	0.363	5.369	0.000	0.223	0.487
Psychological ownership → revitalization intention	0.098	1.355	0.175	–0.042	0.238
Perceived output quality → revitalization intention	0.384	6.700	0.000	0.276	0.497

Table 8 reports the full-sample mediation results. Psychological ownership significantly mediated the relationships between AI process characteristics and cultural heritage revitalization intention. Perceived output quality also significantly mediated the relationships between AI-generated content characteristics and cultural heritage revitalization intention. These results support H6 and H7 in the full sample.

**TABLE 8:** Mediation effects for the full sample.

Indirect path	Effect	SD	*T*-value	*P*-value	95% CI
Algorithmic autonomy → psychological ownership → revitalization intention	–0.098	0.026	3.783	0.000	[–0.150, –0.046]
Interaction transparency → psychological ownership → revitalization intention	0.063	0.020	3.148	0.002	[0.027, 0.106]
Interaction depth → psychological ownership → revitalization intention	0.063	0.021	2.979	0.003	[0.026, 0.109]
Cultural authenticity → perceived output quality → revitalization intention	0.078	0.021	3.670	0.000	[0.041, 0.124]
Visual complexity → perceived output quality → revitalization intention	0.072	0.021	3.439	0.001	[0.035, 0.116]

### 4.4 Measurement invariance and permutation-based multi-group analysis

Before comparing path coefficients between experienced and early career designers, measurement invariance was assessed using the MICOM procedure. As shown in Table 9, configural invariance was established for all constructs. Full measurement invariance was supported for seven constructs: AA, IT, CA, VC, PO, OQ, and RI. The only exception was ID, for which compositional invariance was not established at Step 2. Accordingly, the group comparison involving the ID - > PO path is reported but interpreted with caution.

**TABLE 9:** MICOM results for measurement invariance.

Construct	Step 1 configural invariance	Original c	5% Quantile	Step 2 compositional invariance	Equal means	Equal variances	Conclusion
AA	Yes	0.996730	0.994509	Yes	Yes	Yes	Full invariance
IT	Yes	0.993401	0.992540	Yes	Yes	Yes	Full invariance
ID	Yes	0.989808	0.989869	No	Yes	Yes	Not established at Step 2
CA	Yes	0.993661	0.988103	Yes	Yes	Yes	Full invariance
VC	Yes	0.999420	0.991623	Yes	Yes	Yes	Full invariance
PO	Yes	0.998989	0.998776	Yes	Yes	Yes	Full invariance
OQ	Yes	0.997210	0.996690	Yes	Yes	Yes	Full invariance
RI	Yes	0.999621	0.996187	Yes	Yes	Yes	Full invariance

Compositional invariance is established when the original correlation is greater than or equal to the 5% permutation quantile. Full invariance is concluded when compositional invariance, equal means, and equal variances are supported.

Permutation-based multi-group analysis was then conducted to examine structural differences between the two designer groups. As reported in Table 10, psychological ownership had a significantly stronger effect on revitalization intention among experienced designers (Δβ = 0.268, *p* = 0.015), supporting H8a. By contrast, perceived output quality had a significantly stronger effect on revitalization intention among early career designers (Δβ = –0.224, *p* = 0.016), supporting 10. Visual complexity also had a stronger effect on perceived output quality in the early career group (Δβ = –0.292, *p* = 0.008), indicating that early career designers were more sensitive to visual refinement when evaluating AI-generated outputs. No significant group differences were found for the remaining paths. Overall, experienced designers’ revitalization intention was more closely associated with ownership-based agency, whereas early career designers relied more on output-based evaluation.

**TABLE 10:** Permutation-based multi-group analysis results.

Path	β experienced	β early career	Difference	Permutation p	Conclusion
AA - > PO	–0.431	–0.535	0.104	0.248	Not significant
IT - > PO	0.315	0.297	0.018	0.852	Not significant
ID - > PO	0.312	0.334	–0.022	0.824	Not significant; interpret cautiously
CA - > OQ	0.254	0.298	–0.044	0.715	Not significant
VC - > OQ	0.071	0.363	–0.292	0.008	Significant; stronger in early career group
PO - > RI	0.366	0.098	0.268	0.015	Significant; stronger in experienced group
OQ - > RI	0.161	0.384	–0.224	0.016	Significant; stronger in early career group

Difference was calculated as β experienced—β early career. Negative values indicate stronger effects in the early career group.

### 4.5 Fuzzy-set qualitative comparative analysis (fsQCA)

To complement the linear SEM results, fsQCA was used to examine how different combinations of antecedent conditions led to high cultural heritage revitalization intention. The analysis was conducted in fsQCA 4.1 using the 312 valid cases. All variables were calibrated using three anchors: full membership at the 95th percentile, the crossover point at the 50th percentile, and full non-membership at the 5th percentile (Ragin, 2009). The calibration anchors are reported in Table 11.

**TABLE 11:** Calibration anchors for variables.

Variable type	Variable	Full membership (95th percentile)	Crossover point (50th percentile)	Full non-membership (5th percentile)
Outcome variable	Cultural heritage revitalization intention (Y)	6.6667	4.6667	2.3333
Condition variable	Algorithmic autonomy (X1)	6.8167	5.0000	2.3333
	Interaction transparency (X2)	6.4833	4.6667	2.3333
	Interaction depth (X3)	6.4833	5.0000	2.3333
	Cultural authenticity (X4)	6.6667	5.0000	2.3333
	Visual complexity (X5)	6.3333	4.6667	2.3333
	Psychological ownership (X6)	6.3333	4.6667	2.3333
	Perceived output quality (X7)	6.6667	4.8333	2.3333

The necessity analysis is presented in Table 12. The consistency values for all conditions were below 0.90, indicating that no single condition was necessary for high revitalization intention. This result suggests that high revitalization intention depends on combinations of conditions rather than any single antecedent.

**TABLE 12:** Necessity analysis.

Condition	Consistency	Coverage
Algorithmic autonomy (X1)	0.703379	0.726593
∼Algorithmic autonomy (∼X1)	0.628942	0.620732
Interaction transparency (X2)	0.703659	0.697924
∼Interaction transparency (∼X2)	0.596549	0.613064
Interaction depth (X3)	0.702605	0.734967
∼Interaction depth (∼X3)	0.602448	0.587576
Cultural authenticity (X4)	0.698623	0.705971
∼Cultural authenticity (∼X4)	0.620375	0.625576
Visual complexity (X5)	0.722900	0.715378
∼Visual complexity (∼X5)	0.574780	0.592092
Psychological ownership (X6)	0.697372	0.723997
∼Psychological ownership (∼X6)	0.610717	0.599887
Perceived output quality (X7)	0.731314	0.738272
∼Perceived output quality (∼X7)	0.581308	0.586764

The sufficiency analysis identified five configurations leading to high cultural heritage revitalization intention, as shown in Table 13. The overall solution consistency was 0.889, and the overall solution coverage was 0.445. Algorithmic autonomy appeared as a core condition in all five configurations. Configurations 2 and 3 indicate an ownership-oriented pathway: when psychological ownership and cultural authenticity are present, high revitalization intention can emerge even when output quality or visual complexity is not consistently high. This pattern is consistent with the stronger role of agency among experienced designers. Configurations 1 and 4 indicate a utility-oriented pathway: when visual complexity and perceived output quality are present, revitalization intention can be supported even when psychological ownership or cultural authenticity is less central. This pattern aligns with the instrumental orientation observed among early career designers. Configuration 5 shows a further pathway in which cultural authenticity and psychological ownership jointly support revitalization intention.

**TABLE 13:** Configurational analysis results for cultural heritage revitalization intention.

Condition	Configuration 1	Configuration 2	Configuration 3	Configuration 4	Configuration 5
Algorithmic autonomy (X1)	•	•	•	•	•
Interaction transparency (X2)			•	•	•
Interaction depth (X3)	•	•	•	•	
Cultural authenticity (X4)	•	•	•		•
Visual complexity (X5)	•		•	•	•
Psychological ownership (X6)		•	•	•	•
Perceived output quality (X7)	•	•		•	•
Consistency	0.919964	0.933779	0.937833	0.945763	0.921656
Raw coverage	0.360656	0.358357	0.309714	0.312489	0.314661
Unique coverage	0.040280	0.037981	0.012910	0.015685	0.017857

• = core condition present; blank cells = “do not care.” Overall solution consistency = 0.889 (>0.80); overall solution coverage = 0.445.

Taken together, the fsQCA results show that cultural heritage revitalization intention is produced through multiple combinations of conditions. These findings complement the SEM results by showing how ownership-based agency and utility-based evaluation operate together in different pathways.

### 4.6 LDA evidence from cross-context textual analysis

To assess whether the survey findings were reflected in broader design discourse, this study used cross-context textual analysis as supplementary triangulation. The professional corpus was drawn from publicly available design forums and industry summit interviews, while the public application corpus was collected from public social media tutorials on AI-assisted design. The purpose of this analysis was not to test the causal model a second time, but to examine whether the ownership-based and quality-evaluation orientations appeared in naturally occurring discourse.

Before modeling, non-text entries were removed and the remaining Chinese texts were cleaned by removing numbers, punctuation, special characters, and stop words. Directly identifying information, including usernames and platform-specific identifiers, was removed during cleaning. Word segmentation was conducted using Jieba, and very short tokens were excluded. The cleaned texts were converted into bag-of-words vectors with Gensim. Terms with very low or very high frequency were filtered using no_below = 2 and no_above = 0.7. LDA models were then estimated with Gensim, and topic coherence was calculated to assess interpretability. The coherence scores were 0.62 for the expert corpus and 0.59 for the public corpus, indicating acceptable topic separation. Tables 14, 15 report the topic distributions.

**TABLE 14:** LDA topic modeling results for the expert corpus.

Topic	Share	Representative keywords	Representative excerpt
Topic 1: industrialized design system	0.2500	Sketch, certainty, rendering, design institute, hand drawing, color, ideation, mechanization, industrial system, knowledge system	In basic design teaching, students used to spend considerable effort developing color compositions. Now they can describe their imagined color scheme to AI, and if the prompt is well written, AI can generate a satisfactory composition.
Topic 2: subjective control and design craft	0.1549	Architect, manual work, perspective, subconscious, control, payment, manifestation, technique, tendency, refinement	The traditional paradigm of architectural design has not changed. Designers still need task analysis, site analysis, conceptual sketches, models, and comparison of multiple schemes, but AI tools can assist at each stage.
Topic 3: bodily perception and market recognition	0.2007	Form, scale, viewpoint, contact, gravity, linearity, prompt, target market, recognizability, inspiration	AI does not automatically make a design good, nor does using AI mean that the author lacks ability. The outcome still needs to reflect human agency and the designer’s purpose.
Topic 4: cross-disciplinary formal creation	0.2007	Packaging, invariance, deduction, architecture, detail, shape, fuselage, creativity, floor plan, assisted design	Judgment remains the most important issue. For students, this era requires cross-disciplinary learning and stronger control over expression, thinking, and detail. For architects, AI may become as ordinary as a pen.
Topic 5: digital design logic	0.1937	Construction, angle, logical relation, computer, user, judgment, calculator, designer, voice input, design-driven	The design logic folds traditional cultural scenes into a new form. If we do not understand that logic, turning such culture only into temples or museums may not preserve it, but rather weaken it.

**TABLE 15:** LDA topic modeling results for the public corpus.

Topic	Share	Representative keywords	Representative excerpt
Topic 1: creative ideation and visual tuning	0.2179	Modification, tone, probability, sketch, shape, inspiration, text effect, template, color matching, visualization	When suitable materials cannot be found, users can first draw a rough sketch with simple shapes and then ask AI to generate materials based on the reference image, depth, and target theme.
Topic 2: cultural-creative visuals and storyboard composition	0.1667	Storyboard, icon, cultural heritage, design sense, image generation, color block, shot, geometry, cultural-creative product, repetition	A complete set of storyboard images can be produced in less than a minute, with visual consistency close to that of a professional team. AI can also extract layouts and turn image elements into editable materials.
Topic 3: detailed refinement and parameter control	0.2949	Detail, parameter, layout, effect, product image, prompt, marker, copywriting, fine-tuning, refinement	Taking GPT-4 as an example, users need to understand parameters such as temperature. Lower values produce more stable and repeatable results, while higher values increase randomness and suit more creative tasks.
Topic 4: commercial presentation and output production	0.3205	Rendering, poster, decomposition, portfolio, script, mockup, splitting, commercial advertisement, canvas, color	Users can upload a product image to generate model-in-hand product visuals without complex prompts. With added style descriptions, AI can quickly produce posters, product layouts, and commercial advertising images.

The expert context identified five core topics, including the industrialized design system, which accounted for 25.0%, and cross-disciplinary formal creation and bodily perception, each accounting for about 20.1%. These topics suggest that experienced experts give considerable attention to creative control, judgment, and professional agency when using AI in design. By contrast, the public application context produced four independent topics: commercial presentation and output production (32.1%), detailed refinement and parameter control (29.5%), creative ideation and visual tuning (21.8%), and cultural-creative visuals and storyboard composition (16.7%). These patterns are consistent with the presence of two discourse orientations: one centered on agency and design judgment, and the other centered on output completion and practical use.

## 5 Discussion

### 5.1 AI process cues and psychological ownership

RQ1 examined how AI process cues affect designers’ psychological ownership. Algorithmic autonomy reduced psychological ownership among both experienced and early career designers (β = –0.431; β = –0.535). This result is consistent with Pierce et al. (2001) argument that perceived control is a source of psychological ownership. It also supports Hermann (2025) view that generative AI can dilute creators’ sense of ownership. Messer (2024) further showed that evaluations of both the work and the creator decline when AI enters the implementation stage and makes key decisions such as layout. This study extends that logic to cultural heritage design. Algorithmic autonomy weakens designers’ perceived ownership of cultural interpretation, rather than general satisfaction with the tool. Interaction transparency (β = 0.315; β = 0.297) and interaction depth (β = 0.312; β = 0.334) both enhanced psychological ownership. This finding aligns with Kizilcec (2016) conclusion that algorithmic transparency strengthens understanding and trust. Multi-round adjustment allows designers to embed their judgment, selection, and revision into AI outputs. It therefore restores a subjective sense of ownership over the work. The mediation results further support this interpretation. Algorithmic autonomy reduced revitalization intention through psychological ownership (indirect effect = –0.098), while transparency and interaction depth increased revitalization intention through the same path (indirect effects = 0.063).

### 5.2 AI Content cues and perceived output quality

RQ2 focused on how AI-generated content affects perceived output quality. Cultural authenticity significantly predicted perceived output quality in both groups (β = 0.254; β = 0.298). This result is consistent with research on cultural heritage authenticity by Kolar and Zabkar (2010) and Farrelly et al. (2019). In cultural heritage design, quality depends on the validity of symbolic origins, cultural context, and meaning translation. It cannot be reduced to image refinement. The effect of visual complexity was more differentiated. Visual complexity significantly influenced early career designers’ quality judgments (β = 0.363), but its effect was not significant among experienced designers (β = 0.071). This finding partly supports Guo et al. (2018) view that visual complexity can serve as a cue for aesthetic quality. It also clarifies the boundary of this effect. Early career designers are more likely to associate rich detail and layered composition with professional completeness. Experienced designers are less likely to equate visual complexity with cultural appropriateness. Bellaiche et al. (2023) argued that evaluations of AI art depend strongly on authenticity and perceived human input. This study further shows that, in cultural heritage design, visual appeal has stable professional value only when cultural authenticity is established.

### 5.3 Professional experience and divergent revitalization pathways

RQ3 tested whether professional experience changes the effects of psychological ownership and perceived output quality on revitalization intention. Experienced designers’ revitalization intention depended more on psychological ownership (β = 0.366). Early career designers’ intention depended more on perceived output quality (β = 0.384). The permutation-based multi-group analysis also showed that the psychological ownership path was stronger in the experienced group (Δβ = 0.268, *p* = 0.015), while the output quality path was stronger in the early career group (Δβ = –0.224, *p* = 0.016). This result is consistent with Ahmed et al. (2003) work on differences between novice and expert design strategies. It also aligns with Ozkan and Dogan (2013) finding that experts rely more on structural judgment, while novices rely more on visible cues. Atkinson and Barker (2023) found that the role of generative AI in design tasks varies by creator experience and task stage. This study provides similar evidence in the context of cultural heritage design. Experienced designers focus on whether AI preserves their authority in cultural judgment. Early career designers focus on whether AI produces usable, complete, and presentable design resources. The fsQCA results further complement the linear model. No single condition reached the necessity threshold of 0.90, and the overall solution consistency was 0.889. This indicates that high revitalization intention arises from multiple sufficient configurations, rather than from a universal effect of any single AI characteristic. The behavioral science contribution of this study lies in revealing differentiated psychological mechanisms in AI-assisted cultural revitalization. Designers with more experience act through an ownership logic. Designers with less experience act through a quality-utility logic.

### 5.4 Theoretical and practical implications

This research reframes AI-assisted cultural heritage design as an issue of designers’ evaluation, subjective experience, and behavioral intention, extending the discussion beyond tool performance. Prior studies have mainly examined whether AI improves generation efficiency, expands creative possibilities, or enhances design outputs. They have offered limited explanation of why designers are willing to continue using AI for cultural heritage revitalization. The findings show that AI involvement affects designer behavior through multiple paths. It triggers judgments of both the collaborative process and the generated content. This study therefore proposes a dual-path framework that is closer to cultural design practice. This distinction helps clarify the study’s conceptual contribution: designers evaluate whether they retain a position in creative and cultural interpretation, and whether AI-generated outputs can become credible resources for cultural translation. Designers evaluate whether they retain a position in creation and cultural interpretation. They also evaluate whether AI outputs can support further design translation.

The moderating role of professional experience further shows that this evaluative mechanism varies across designer groups. Experienced designers place greater emphasis on agency and judgment authority. Early career designers place greater emphasis on output usability and completeness. This finding enriches AI collaboration research by clarifying differences among creators. It further extends design cognition research by showing how experience shapes the relative weight of process-based agency and output-based quality evaluation in culturally sensitive design tasks. It also suggests that willingness to engage in cultural heritage revitalization captures designers’ behavioral orientation toward cultural translation and sustained practice more directly than general technology use intention. By combining SEM, MGA, fsQCA, and LDA text analysis, this study demonstrates the complementary value of linear paths, group differences, condition configurations, and real-context discourse. It also offers a more robust methodological approach for studying AI involvement in complex cultural practices.

The findings also provide practical implications for cultural institutions, design teams, AI platforms, and design education. AI should be deployed as more than a fast image-generation tool. A uniform workflow is also unlikely to serve all designers effectively. For experienced designers, systems should provide clear generation logic, traceable revision processes, and sufficient space for fine-grained intervention. These functions allow them to maintain cultural judgment and design oversight in AI collaboration. For early career designers and students, platforms should provide more reliable cultural materials, clearer symbolic explanations, and more stable output quality. These supports can help them transform traditional elements into design solutions with further development potential.

Cultural institutions can play a stronger role in setting the conditions for AI-assisted heritage revitalization. They can build AI-oriented heritage material libraries, pattern genealogies, case prompts, and cultural semantic descriptions. These resources should include provenance, historical context, symbolic meanings, adaptation boundaries, and restrictions on culturally sensitive use. These resources can reduce symbolic collage and contextual misinterpretation during generation. Institutions can also develop authenticity guidelines, support curator-designer collaboration, and introduce cultural validation procedures for AI-generated outputs before they enter exhibitions, products, or public communication. These arrangements help designers judge whether generated outputs remain culturally grounded and suitable for further development. Design education should also move beyond prompt operation training. It should cultivate students’ ability to judge whether AI outputs have cultural grounding, translation value, and contemporary expressive capacity. Effective use of AI in cultural heritage design therefore depends on easier access to generative tools, reliable cultural resources, professional judgment, and validation mechanisms that help designers assess whether AI-generated outputs are culturally grounded.

### 5.5 Limitations and future research

Several limitations should be acknowledged. The study treats AI-assisted cultural heritage design as a broad research context rather than distinguishing among heritage types, design media, or institutional settings. Textiles, architecture, artifacts, intangible heritage practices, and digital exhibitions may place different demands on cultural authenticity, visual complexity, and design translation. Institutional arrangements, including digital heritage libraries, authenticity guidelines, curatorial participation, and cultural validation procedures, may also shape how designers interpret AI process and content cues. Future research could compare AI collaboration across different cultural media and examine these institutional arrangements as boundary conditions.

The analysis also relies on designers’ retrospective evaluations of prior AI collaboration experiences. Such evaluations are useful for capturing professional judgment, but they cannot fully trace how psychological ownership, perceived output quality, or trust in AI changes over time. Longitudinal studies, diary-based designs, or tracked design tasks could offer a more dynamic account of how designers adjust their sense of creative control and cultural judgment through repeated AI use.

The distinction between experienced and early career designers was based on years of design-related experience and professional background. Although this classification helps identify group differences, it remains a simplified proxy for design expertise. Future studies could incorporate project complexity, portfolio quality, cultural heritage knowledge, AI proficiency, and design specialization to capture expertise more precisely. Finally, although the study combines survey data, MGA, fsQCA, and LDA text analysis, its overall design remains mainly observational. Experimental work could manipulate key AI process and content cues to test more rigorously how AI involvement shapes designers’ psychological evaluations and willingness to engage in cultural heritage revitalization.

## 6 Conclusion

Based on the S-O-R framework, this study developed a dual-path moderated mediation model. Using a sample of 312 designers with experience in generative AI-assisted cultural heritage design, the study verified two mechanisms. AI process cues influence revitalization intention through psychological ownership. AI-generated content cues influence revitalization intention through perceived output quality. Professional experience further changes the relative strength of these two paths. Experienced designers place greater emphasis on ownership-oriented agency, while early career designers place greater emphasis on output-oriented quality judgment. The fsQCA and LDA results also show that high revitalization intention is shaped by configurations of multiple conditions and their combined role in design contexts.

These findings show that AI-assisted cultural heritage design extends beyond technical efficiency and visual generation capacity. The key issue is whether AI helps designers retain cultural judgment authority, develop psychological attachment to collaborative outcomes, and identify the cultural value and translation potential of generated content. This study links AI collaboration research with cultural heritage revitalization and clarifies how new technologies affect cultural representation, design judgment, and sustained dissemination. The value of generative AI in cultural design lies in supporting designers as they translate traditional cultural elements into design expressions that are understandable, usable, and sustainable in contemporary contexts. In the future, AI platforms, cultural institutions, and design education should optimize collaboration mechanisms around this goal, so that AI can serve as a collaborative medium for cultural translation and sustainable cultural practice.

## Data Availability

The original contributions presented in the study are included in the article/supplementary material, further inquiries can be directed to the corresponding author.
